# The immunologic Warburg effect: Evidence and therapeutic opportunities in autoimmunity

**DOI:** 10.1002/wsbm.1486

**Published:** 2020-02-27

**Authors:** Michael D. Kornberg

**Affiliations:** ^1^ Department of Neurology Johns Hopkins University School of Medicine Baltimore Maryland USA

**Keywords:** aerobic glycolysis, autoimmune disease, immunology, immunometabolism, Warburg effect

## Abstract

Pro‐inflammatory signals induce metabolic reprogramming in innate and adaptive immune cells of both myeloid and lymphoid lineage, characterized by a shift to aerobic glycolysis akin to the Warburg effect first described in cancer. Blocking the switch to aerobic glycolysis impairs the survival, differentiation, and effector functions of pro‐inflammatory cell types while favoring anti‐inflammatory and regulatory phenotypes. Glycolytic reprogramming may therefore represent a selective vulnerability of inflammatory immune cells, providing an opportunity to modulate immune responses in autoimmune disease without broad toxicity in other tissues of the body. The mechanisms by which aerobic glycolysis and the balance between glycolysis and oxidative phosphorylation regulate immune responses have only begun to be understood, with many additional insights expected in the years to come. Immunometabolic therapies targeting aerobic glycolysis include both pharmacologic inhibitors of key enzymes and glucose‐restricted diets, such as the ketogenic diet. Animal studies support a role for these pharmacologic and dietary therapies for the treatment of autoimmune diseases, and in a few cases proof of concept has been demonstrated in human disease. Nonetheless, much more work is needed to establish the clinical safety and efficacy of these treatments.

This article is categorized under:

Biological Mechanisms > Metabolism
Translational, Genomic, and Systems Medicine > Translational Medicine
Biological Mechanisms > Cell Signaling

## Graphical abstract

The immunologic Warburg effect represents a potential target for treating autoimmune diseases, through both pharmacologic and dietary strategies. The mechanisms explaining the requirement for aerobic glycolysis in immune activation remain only partially understood.

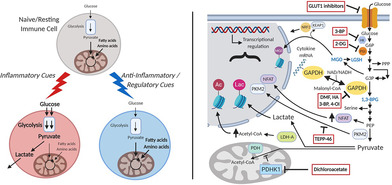

## 1 INTRODUCTION

In the 1920s, Otto Warburg made the seminal observation that tumor cells dramatically upregulate glycolysis compared with surrounding normal tissue, with increased fermentation of pyruvate to lactate rather than oxidation in mitochondria even in the presence of oxygen (Warburg, 1925). This peculiar form of energy metabolism, termed “aerobic glycolysis” and subsequently the “Warburg effect,” has long been proposed to represent a selective vulnerability of cancer cells amenable to therapeutic intervention.

**Figure 1:**
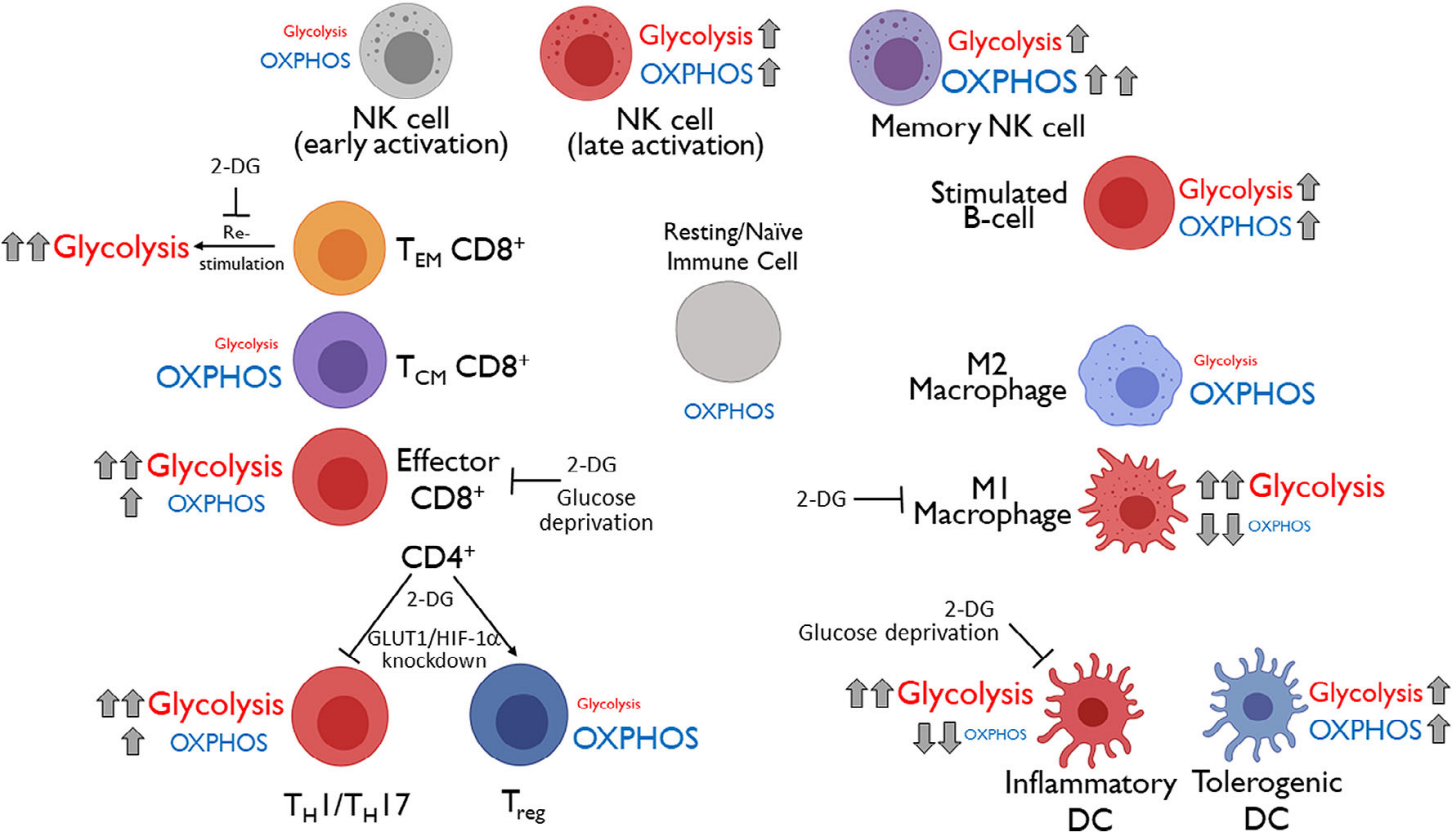
Glycolytic reprogramming is conserved among inflammatory immune subsets. Glycolytic upregulation, including the increased lactate production in the presence of oxygen that defines aerobic glycolysis, occurs following inflammatory activation of cells from both myeloid and lymphoid lineage. In contrast, regulatory and/or anti‐inflammatory immune subsets generally rely on oxidative energy metabolism. Inhibiting glycolysis through genetic or pharmacologic measures prevents inflammatory immune activation, including the differentiation and effector functions of inflammatory cells, while promoting differentiation of regulatory subsets. 2‐DG, 2‐deoxy‐d‐glucose

Although Warburg himself (Warburg, Gawehn, & Geissler, 1958) and several other groups in the 1960s and 1970s (Cooper, Barkhan, & Hale, 1963; Culvenor & Weidemann, 1976; Hedeskov, 1968; Roos & Loos, 1970) observed a similar metabolic switch to aerobic glycolysis in activated leukocytes, the Warburg effect was widely considered unique to cancer biology until the early 2000s. At that time, seminal work by several groups demonstrated that immune challenge and activation of naïve lymphocytes produces metabolic reprogramming from oxidative to Warburg physiology (Cham, Driessens, O'Keefe, & Gajewski, 2008; Cham & Gajewski, 2005; Frauwirth et al., 2002). In the intervening years, these observations were extended to other immune cell types of both myeloid and lymphoid lineages, and within both the innate and adaptive immune systems (Kelly & O'Neill, 2015; Klein Geltink, Kyle, & Pearce, 2018; O'Neill, Kishton, & Rathmell, 2016; Pearce & Pearce, 2013; Figure 1). The past 15 years have seen an explosion of interest in the role of metabolism in immune regulation, as cellular metabolic pathways previously thought to serve housekeeping functions have been shown to dynamically respond to external signals and causally regulate immune activation, differentiation, and effector function. The result has been the burgeoning field of “immunometabolism” and excitement regarding the possibility of targeting metabolism to modulate the immune response in human disease (Patel, Leone, Horton, & Powell, 2019).

Metabolic regulation of the immune response is much more complex than the simple balance between glycolysis and oxidative phosphorylation (OXPHOS), with a wide and growing array of metabolic pathways interacting to impact immune function (O'Neill et al., 2016; Patel et al., 2019). And just as Warburg physiology is not unique to cancer cells, it extends beyond immunology as well—recent studies have identified important roles for aerobic glycolysis in other proliferative cell types, such as pluripotent stem cells (Folmes et al., 2011; Kondoh et al., 2007) and angiogenic endothelial cells (De Bock et al., 2013). Nonetheless, as a common characteristic among inflammatory cell types and a critical regulator of immune function, aerobic glycolysis remains an attractive therapeutic target for treating disorders of immune dysregulation (e.g., autoimmune diseases) without broad toxicity in normal, differentiated tissues that rely on oxidative metabolism. And unlike cancer, in which the therapeutic effect must be complete, the treatment of autoimmune diseases requires only modulation of the immune response. Our own recent work found that dimethyl fumarate, a derivative of the Krebs cycle metabolite fumarate used to treat autoimmune diseases such as psoriasis and multiple sclerosis (MS) in humans, produces anti‐inflammatory effects via inhibition of aerobic glycolysis (Kornberg et al., 2018). This work provided proof of concept that targeting aerobic glycolysis is a viable strategy for controlling autoimmunity.

In this review, I will first survey the evidence supporting the critical role of aerobic glycolysis in immune fate and function, highlighting multiple cell types of both lymphoid and myeloid lineage. I will then discuss our current knowledge and the many unanswered questions surrounding the mechanistic requirement for glycolytic reprogramming, that is, why it is necessary for immune activation. Finally, I will review current pharmacologic and dietary efforts to target glycolysis as a therapeutic strategy, focusing on autoimmune disease.

## 2 EVIDENCE FOR THE IMPORTANCE OF AEROBIC GLYCOLYSIS IN IMMUNE REGULATION

### 2.1 T lymphocytes

The earliest insights into the importance of glycolytic reprogramming in the immune response were derived from studies in T lymphocytes, of both CD8 and CD4 lineage. In 2002, Frauwirth and colleagues in the laboratory of Craig Thompson discovered that upregulation of glycolysis in T cells is a coordinated response to immune activation, with CD28 co‐stimulation producing increased glucose transporter expression, glucose uptake, and glycolysis via phosphatidylinositol 3‐kinase (PI3K). They also made the observation that glucose was preferentially metabolized to lactate in this setting rather than oxidized in mitochondria—the hallmark of Warburg physiology. Soon after, Cham and colleagues (Cham et al., 2008; Cham & Gajewski, 2005) showed that this coordinated glycolytic reprogramming is required for CD8 effector functions such as cytokine transcription and cytolytic activity, which were inhibited by glucose deprivation and the glycolysis inhibitor 2‐deoxy‐d‐glucose (2‐DG). Several groups have subsequently extended these seminal observations, showing that enhanced glycolysis is similarly necessary for survival/proliferation, differentiation, and effector functions of CD4 lymphocytes and identifying molecular pathways underlying glycolytic reprogramming in lymphocytes, such as transcriptional regulation by Myc (Wang et al., 2011) and hypoxia‐inducible factor 1α (HIF‐1α; Shi et al., 2011), increased expression of the glucose transporter GLUT1 (Macintyre et al., 2014), and activation of pyruvate dehydrogenase kinase isoform 1 (PDHK1; Gerriets et al., 2015).

T lymphocytes span a wide range of phenotypes and functions, and importantly glycolytic reprogramming and the balance between glycolysis and OXPHOS differentially regulate T cell subsets and inflammatory versus regulatory functions. For instance, pro‐inflammatory effector CD4 lymphocytes such as T helper (T_H_) 1 and T_H_17 cells are characterized by Warburg‐like glycolytic metabolism, requiring aerobic glycolysis for their differentiation and effector functions. Regulatory T (T_reg_) cells on the other hand, which suppress inflammatory responses and promote tolerance, engage glycolytic metabolism during initial activation and proliferation but subsequently exhibit oxidative metabolism dependent on lipids and pyruvate and become independent of glucose (Angelin et al., 2017; Gerriets et al., 2016;Michalek et al., 2011; Shi et al., 2011). Preventing glycolytic upregulation via glucose deprivation, GLUT1 or HIF‐1α deficiency, or 2‐DG shifts the balance between these subsets, preventing T_H_1/T_H_17 differentiation and reciprocally favoring T_reg_ development both in vitro and in vivo (Michalek et al., 2011; Shi et al., 2011). Activation of T_reg_ cells with toll‐like receptor (TLR) agonists or forced expression of Glut1 increases glycolysis and proliferation but inhibits their anti‐inflammatory suppressive functions, which depend on oxidative metabolism driven by the transcription factor Foxp3 (Gerriets et al., 2016).

Bioenergetic profiles similarly distinguish effector from central memory T (T_CM_) lymphocytes, which has been principally studied in CD8 cells. Whereas effector CD8 lymphocytes display Warburg physiology, long‐lived T_CM_ CD8 cells primarily utilize fatty acid oxidation (O'Sullivan et al., 2014; Pearce et al., 2009; Pollizzi et al., 2015; Sukumar et al., 2013; van der Windt et al., 2012). Inhibition of glycolysis promotes and OXPHOS inhibition impairs T_CM_ development. Upon re‐stimulation, memory CD8 cells once again engage aerobic glycolysis to perform effector functions (Gubser et al., 2013). The ability to shift T lymphocyte fate and function from pro‐inflammatory to regulatory or memory phenotypes by inhibition of glycolysis underlies the potential of targeting aerobic glycolysis in autoimmune disease.

As in the case of cancer, it is important to note that the balance between glycolysis and OXPHOS is not all or none. Although glycolysis is disproportionately upregulated following pro‐inflammatory stimulation of naïve T lymphocytes, with substantial lactate production consistent with aerobic glycolysis, OXPHOS increases as well (but to a lesser extent) and serves critical functions. For instance, reactive oxygen species derived from the electron transport chain (ETC) are essential for T lymphocyte activation (Sena et al., 2013), and inhibition of OXPHOS with oligomycin prevents initial T lymphocyte activation and proliferation (Chang et al., 2013). Aerobic glycolysis appears to be dispensable for the earliest stage of T lymphocyte activation (Chang et al., 2013; Tan et al., 2017), whereas T cell‐specific knockdown of ETC components limits early activation and expansion (Tan et al., 2017; Tarasenko et al., 2017). Mitochondrial serine metabolism is critical for T cell activation (Ron‐Harel et al., 2016), and mitochondrial biogenesis induced by PGC1α is essential for effector T cell antitumor immunity (Scharping et al., 2016). As such, mitochondrial metabolism and OXPHOS serve important energetic, anabolic, and signaling roles in effector T lymphocytes independent of glycolysis.

### 2.2 Dendritic cells

As the primary antigen presenting cells of the peripheral immune system, dendritic cells (DCs) link the innate and adaptive arms of immunity and regulate lymphocyte activation and differentiation. Upon exposure to antigens and other external signals, DCs can adopt pro‐inflammatory or tolerogenic phenotypes and subsequently migrate from peripheral tissues to draining lymph nodes. Stimulation of pattern recognition receptors, such as TLRs, by signals such as lipopolysaccharide (LPS) promote inflammatory DC activation, inducing production of pro‐inflammatory chemokines and cytokines. Resting DCs exhibit oxidative metabolism. Ligation of TLRs induces a drastic increase in glycolysis associated with lactate production, consistent with Warburg physiology (Krawczyk et al., 2010). This glycolytic reprogramming is required for both DC activation and survival (Everts et al., 2012, 2014; Krawczyk et al., 2010), which are sensitive to glucose deprivation and 2‐DG. Glycolytic upregulation is similarly critical for DC migration to lymph nodes (Guak et al., 2018; Liu et al., 2019). Similar to T lymphocytes, glycolytic upregulation in DCs depends on PI3K and AKT signaling (Everts et al., 2014; Krawczyk et al., 2010), as well as HIF‐1α (Guak et al., 2018; Liu et al., 2019). Unlike T lymphocytes, inflammatory activation of DCs leads to a drastic reduction in OXPHOS as a result of inhibition by nitric oxide (NO), and glycolytic upregulation is therefore required not only to support effector functions but also to maintain ATP levels to support survival (Everts et al., 2012).

The metabolic phenotype of tolerogenic DCs has been less studied, but work in human cells found that tolerogenic DCs increase both glycolysis and OXPHOS, with OXPHOS driven by fatty acid oxidation playing a critical role in maintaining a tolerogenic phenotype (Malinarich et al., 2015).

### 2.3 Macrophages

Metabolic reprogramming toward aerobic glycolysis plays a critical role in macrophage activation and polarization—a concept that was “re‐discovered” within the past 15 years after initial descriptions dating back to 1970 (Hard, 1970). Similar to other immune cells, macrophages take on distinct phenotypes in response to external signals. Inflammatory signals, such as interferon gamma (IFNγ), LPS, and other TLR agonists, produce classically activated (or “M1”) macrophages, which are bactericidal but also perpetuate inflammation and promote tissue injury by producing cytokines and toxic oxygen and nitrogen free radicals. Alternatively activated (or “M2”) macrophages, on the other hand, are induced by signals such as interleukin (IL)‐4 and IL‐13 and promote resolution of inflammation and tissue repair. M1 macrophages upregulate glycolysis and display Warburg physiology, while M2 macrophages maintain glycolysis at levels comparable to unstimulated cells and depend on OXPHOS (Rodríguez‐Prados et al., 2010; Vats et al., 2006). In keeping with the paradigm described above for T lymphocytes and DCs, aerobic glycolysis is required for inflammatory functions of M1 macrophages, which can be blocked by 2‐DG (Tannahill et al., 2013) and augmented by overexpression of GLUT1 (Freemerman et al., 2014). Similar to DCs, M1 macrophages downregulate OXPHOS, largely due to inhibition by NO. Several mechanisms underlying glycolytic reprogramming following TLR activation have been described, as reviewed elsewhere (Kelly & O'Neill, 2015), including activation of the mechanistic target of rapamycin (mTOR) and HIF‐1α pathways, increased expression of ubiquitous‐type phosphofructokinase 2 (u‐PFK2), and downregulation of AMP‐activated protein kinase (AMPK).

In contrast to inflammatory M1 macrophages, M2 macrophages require OXPHOS for their polarization and tissue repair functions, with dependence on PPARγ‐coactivator‐1β (PGC‐1β)‐induced fatty acid oxidation and mitochondrial biogenesis (Vats et al., 2006) and regulation by efferocytic metabolites (Zhang, Weinberg, et al., 2019). This bioenergetic dichotomy offers additional targets for manipulating macrophage fate and function.

### 2.4 B lymphocytes

The role of glycolysis, and metabolism more generally, has been less studied in B lymphocyte biology, although this is a rapidly advancing field of research. Similar to naïve T lymphocytes, naïve B lymphocytes are metabolically quiescent but appear to rely on OXPHOS for their energy needs, at least in vitro (Kunisawa et al., 2015). Upon activation, B lymphocytes were found to undergo a more balanced increase in lactate production and oxygen consumption compared to T lymphocytes, although glycolytic upregulation is required for proliferation and antibody production (Akkaya et al., 2018; Caro‐Maldonado et al., 2014; Jayachandran et al., 2018; Jellusova et al., 2017). Anergic B lymphocytes remain metabolically quiescent (Caro‐Maldonado et al., 2014). The metabolic characteristics and requirements of long‐lived plasma cells and immunosuppressive regulatory B lymphocytes remain incompletely understood.

### 2.5 Natural killer cells

Natural killer (NK) cells are lymphoid cells that influence immune responses through both direct and indirect mechanisms, displaying cytolytic functions and producing inflammatory cytokines such as IFNγ. They are generally considered part of the innate immune system given their rapid responsiveness and lack of antigen specificity, with primary roles in antiviral and anticancer responses, although they regulate the adaptive immune response through interactions with T lymphocytes (Campbell & Hasegawa, 2013; Crouse, Xu, Lang, & Oxenius, 2015). Recently, NK cells have themselves been shown to possess adaptive characteristics, with the discovery of long‐lived subsets displaying immunologic memory (Cooper et al., 2009; Gamliel et al., 2018; O'Leary, Goodarzi, Drayton, & von Andrian, 2006; Sun, Beilke, & Lanier, 2009).

Recent work has identified unique interactions between metabolism (including glycolytic reprogramming) and NK cell function. Interestingly, resting NK cells, which maintain low levels of both glycolysis and OXPHOS, do not exhibit major changes in metabolism during the first several hours of stimulation despite rapidly taking on effector functions (Keppel, Saucier, Mah, Vogel, & Cooper, 2015). Longer periods of stimulation, on the other hand, produce substantial increases in both aerobic glycolysis and OXPHOS that are required for NK cell proliferation and effector functions (Donnelly et al., 2014; Keating et al., 2016; Mah et al., 2017; Marçais et al., 2014; Viel et al., 2016). NK cells can be divided into CD56^bright^ and CD56^dim^ populations, which differ with regard to responsiveness to cytokines versus receptor ligation and the kinetics with which they become cytotoxic. These populations also appear to differ metabolically, with CD56^bright^ cells undergoing greater metabolic reprogramming (Jensen, Potempa, Gotthardt, & Lanier, 2017; Keating et al., 2016). Memory NK cells appear to share some metabolic characteristics with memory T lymphocytes. Adaptive, memory‐like NK cells from human cytomegalovirus (CMV) seropositive donors exhibited increased OXPHOS and mitochondrial membrane potential compared to canonical NK cells (Cichocki et al., 2018). In mice, clearance of dysfunctional mitochondria via mitophagy was required for successful NK cell contraction‐to‐memory phase transition after CMV infection (O'Sullivan, Johnson, Kang, & Sun, 2015).

Some aspects of glycolytic reprogramming are conserved in NK cells compared to other immune subsets, such as dependence on mTOR complex 1 (mTORC1) and Myc (Donnelly et al., 2014; Loftus et al., 2018; Marçais et al., 2014). NK cell metabolism is unique in other respects. For instance, glycolytic upregulation occurs independent of HIF‐1α (Loftus et al., 2018). Even more interestingly, unlike T lymphocytes that fuel OXPHOS via glutamine entering the Krebs cycle, OXPHOS in activated NK cells is supported by pyruvate metabolized within mitochondria via the citrate–malate shuttle, bypassing the Krebs cycle altogether (Assmann et al., 2017). Transcriptional regulation of the citrate‐malate shuttle occurs through a novel role of sterol regulatory element‐binding protein (SREBP), which is classically considered a regulator of fatty acid and cholesterol synthesis.

## 3 EVOLVING MECHANISTIC INSIGHTS INTO GLYCOLYTIC REGULATION OF IMMUNE FUNCTION

Although the causal role of glycolytic reprogramming in determining immune cell fate and function is well established, the precise mechanisms underlying the necessity of aerobic glycolysis remain incompletely understood. Similar questions as to *why* aerobic glycolysis confers advantage still exist in cancer biology as well, as reviewed by Liberti and Locasale (2016). In both cancer biology and immunology, several theories are widely accepted. For instance, it has been proposed that aerobic glycolysis supports proliferative or anabolic states by providing carbon substrates for the many pathways that branch from glycolysis and provide biomass building blocks, such as the pentose phosphate pathway (important for nucleotide and lipid synthesis) and the de novo serine synthesis pathway. Alternatively, a widely cited concept is that aerobic glycolysis provides a greater rate of ATP generation to support growth and anabolism, with the kinetic advantage of glycolysis over OXPHOS outweighing its decreased efficiency in terms of moles of ATP produced per unit of glucose. While both of these proposals have biological plausibility and a degree of experimental support, they have shortcomings as well. For instance, under Warburg conditions most of the carbon that enters cells as glucose is excreted as lactate rather than used for biosynthesis; in stimulated thymocytes, macromolecular synthesis accounted for only 7% of glucose uptake (Hume, Radik, Ferber, & Weidemann, 1978). Although this inefficiency may have paradoxical advantages, such as offering more precise control of biosynthetic pathways (Lunt & Vander Heiden, 2011), these advantages remain hypothetical. Similarly, it has never been definitively shown that ATP levels are limiting either among tumor cells or activated immune cells (Locasale & Cantley, 2011; Lunt & Vander Heiden, 2011), suggesting that a higher rate of ATP production might not explain the necessity of aerobic glycolysis.

**Figure 2:**
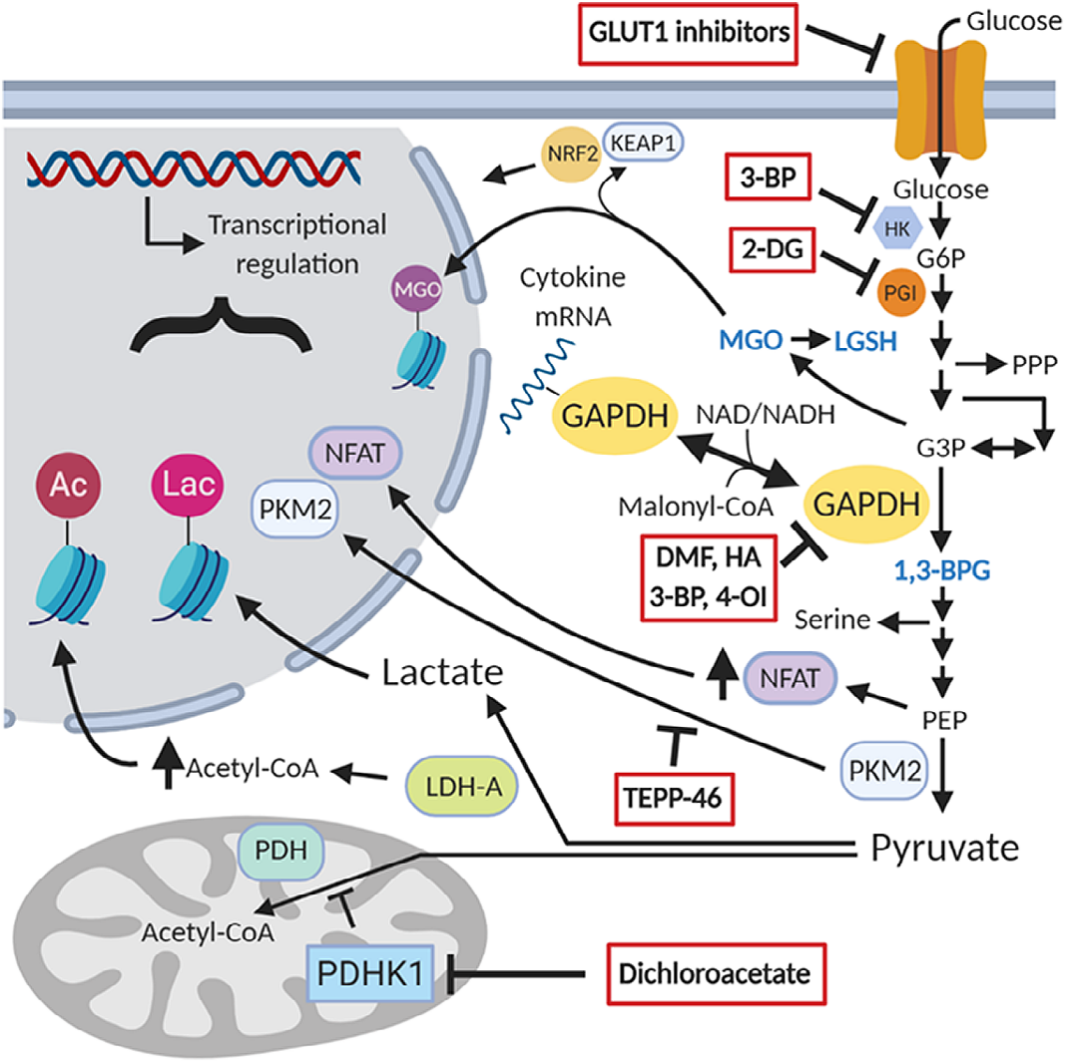
Aerobic glycolysis in immune activation: Mechanisms and pharmacologic targets. The mechanisms by which aerobic glycolysis regulates inflammatory immune functions are still being elucidated. Several currently described transcriptional and posttranscriptional mechanisms of immune regulation are depicted above. Glycolysis‐related metabolites that directly modify proteins are shown in blue. A number of pharmacologic inhibitors of glycolytic enzymes and associated proteins have demonstrated efficacy without toxicity in animal models of autoimmunity or human autoimmune disease. Some examples are shown here, boxed in red. 2‐DG, 2‐deoxy‐d‐glucose; 3‐BP, 3‐bromopyruvate; 4‐OI, 4‐octyl itaconate; DMF, dimethyl fumarate. HA, heptelidic acid; LGSH, lactoylglutathione; MGO, methylglyoxal

The shortcomings of these conventional concepts suggest that other mechanisms may explain the role of aerobic glycolysis in immunology. As described below, several additional mechanisms have been shown to mediate the effects of aerobic glycolysis on immune cell function, including some that are highly novel and surprising (Figure 2). These mechanisms act at both transcriptional and posttranscriptional levels and often rely on direct signaling functions of glycolytic intermediates themselves. Much more remains to be understood about how glycolytic upregulation supports inflammatory immune function, and no doubt many more mechanisms will be described in the years to come.

### 3.1 Posttranscriptional control of cytokine production

Recent work has linked messenger RNA (mRNA) binding by the glycolytic enzyme glyceraldehyde‐3‐phosphate dehydrogenase (GAPDH) to the role of aerobic glycolysis in inflammatory immune responses. Nagy and Rigby (1995) first demonstrated that GAPDH binds to AU‐rich elements in the 3′ untranslated region of mRNA. They found that GAPDH binds to mRNAs corresponding to a number of inflammatory cytokines in splenocytes, including IFNγ and GM‐CSF. GAPDH‐mRNA binding occurred at the NAD^+^‐binding site of GAPDH and competed with binding to NAD^+^/NADH, suggesting competition with the glycolytic functions of GAPDH. In a seminal study, Chang et al. (2013) subsequently showed that GAPDH binding to IFNγ mRNA represses IFNγ translation in T lymphocytes. Upregulation of glycolysis engages the enzymatic activity of GAPDH, leading to the release and translation of IFNγ mRNA—providing a mechanism that explains (at least in part) the necessity of aerobic glycolysis in T lymphocyte activation. GAPDH was similarly shown to repress translation of tumor necrosis factor‐α (TNFα) mRNA in human monocytes and macrophages, with relief of this repression upon glycolytic reprogramming after LPS exposure (Millet, Vachharajani, McPhail, Yoza, & McCall, 2016). More recently, malonylation of GAPDH by malonyl‐CoA, a citrate‐derived metabolite, was shown to induce dissociation of GAPDH and TNFα mRNA in macrophages, suggesting GAPDH‐mRNA binding might be a regulatory node in immune activation impacted by multiple metabolic pathways (Galván‐Peña et al., 2019).

Interestingly, our own group found that inhibition of GAPDH enzyme activity with either dimethyl fumarate or the more specific compound heptelidic acid (also called koningic acid) prevents inflammatory responses in lymphocytes and macrophages despite slightly *decreasing* GAPDH‐mRNA binding—via modification of a cysteine residue required for both catalytic activity and mRNA binding (Kornberg et al., 2018). This finding suggests that GAPDH enzyme activity, and increased glycolytic flux more generally, support immune functions in ways beyond regulation of GAPDH–mRNA binding alone.

### 3.2 Transcriptional control of inflammatory gene networks and glycolytic intermediates as signaling molecules

Aerobic glycolysis has been shown to regulate transcriptional programs in activated immune cells through both epigenetic and other mechanisms. In activated T_H_1 cells, increased activity of LDH‐A, which catalyzes the conversion of pyruvate to lactate that defines aerobic glycolysis, induces histone acetylation and increased transcription of IFNγ by maintaining levels of acetyl‐CoA (Peng et al., 2016). Glycolytic intermediates themselves can also play direct roles in signaling. For instance, phosphoenolpyruvate enhances calcium‐mediated activation of the pro‐inflammatory transcription factor nuclear factor of activated T cells (NFAT) by directly blocking calcium re‐uptake (Ho et al., 2015). Methylglyoxal (MGO), a highly reactive by‐product of glycolysis generated from glyceraldehyde‐3‐phosphate (G3P) and dihydroxyacetone phosphate (DHAP), is a direct source of non‐enzymatic posttranslational protein modification. MGO has been shown to activate the nuclear factor (erythroid‐derived 2)‐related factor 2 (NRF2) antioxidant signaling pathway via electrophilic modification of kelch‐like ECH‐associated protein 1 (KEAP1), which is known to play important roles in immune functioning (Bollong et al., 2018; Mills et al., 2018). MGO also directly modifies histones (Galligan et al., 2018), although the functional consequences of this modification remain to be determined. Recently, Zhang, Tang, et al. (2019) made the startling discovery that lactate itself directly regulates gene expression in macrophages through posttranslational modification of histones, a process termed “lactylation.” Histone lactylation occurred as a late event following exposure to M1‐polarizing stimuli and served to induce a homeostatic, M2‐like transcriptional program, suggesting that in some cases glycolytic metabolites may act as negative regulators of inflammation, restoring balance after prolonged inflammatory activation. Other glycolytic intermediates known to serve signaling roles, albeit with incompletely understood consequences, are 1,3‐bisphosphoglycerate and MGO‐derived lactoylglutathione, both of which posttranslationally modify lysine residues (Gaffney et al., 2019; Moellering & Cravatt, 2013).

## 4 INHIBITION OF AEROBIC GLYCOLYSIS AS A THERAPEUTIC STRATEGY IN AUTOIMMUNE DISEASE

Underlying the excitement surrounding immunometabolism is the goal of exploiting the metabolic characteristics and vulnerabilities of immune subsets to treat disease, using pharmacologic or dietary approaches to fine‐tune immune responses without broad toxicity. Given the role of glycolytic reprogramming in immune responses, strategies aimed at interfering with aerobic glycolysis have been of major interest. With some exceptions, these strategies remain at a pre‐clinical stage of testing. But the success in animal models of pharmacologic and dietary approaches targeting glucose metabolism, as well as the few proof of concept examples in which drugs targeting bioenergetics have been used in human disease, underscores the clinical potential of this strategy. In this section, I will review the preclinical and clinical evidence for inhibiting glycolysis to treat inflammatory and autoimmune conditions (Figure 2 and Table 1). There is similar interest in manipulating metabolism to *augment* immune responses, for instance in the setting of cancer immunotherapy, but these efforts are beyond the scope of this discussion.

**Table 1:** Studies of glucose‐restricted diets in animal models of autoimmunity and human autoimmune disease

Glucose‐restricted diet	Animal models	Human trials	Human trial outcomes
Ketogenic diet	EAE (Choi et al., 2016; Kim et al., 2012)	MS (Phase I—Brenton et al., 2019; Choi et al., 2016)	Improved patient‐reported health measures Mild decrease in lymphocyte count
Continuous calorie restriction	EAE (Esquifino, Cano, Jiménez, Cutrera, & Cardinali, 2004; Esquifino, Cano, Jimenez‐Ortega, Fernández‐Mateos, & Cardinali, 2007; Piccio, Stark, & Cross, 2008)	MS (Phase I—Fitzgerald et al., 2018)	Improvement in patient‐reported emotional health outcomes
Intermittent fasting or fasting‐mimicking diet	EAE (Choi et al., 2016; Cignarella et al., 2018; Kafami et al., 2010)	MS (Phase I—Choi et al., 2016; Fitzgerald et al., 2018) RA (Phase I—Sköldstam, 1986; Kjeldsen‐Kragh et al., 1991)	MS—Improvements in patient‐reported outcomes (Choi et al., 2016; Fitzgerald et al., 2018) MS—Mild decrease in WBC and lymphocyte count (Choi et al., 2016) RA—improvement in patient‐reported outcomes

Abbreviations: EAE, experimental autoimmune encephalomyelitis; MS, multiple sclerosis; RA, rheumatoid arthritis.

### 4.1 Pharmacologic approaches

A variety of glycolytic inhibitors have been used successfully as systemic therapies in animal models of autoimmunity. 2‐DG, which is phosphorylated by hexokinase but then acts as a competitive inhibitor of phosphoglucoisomerase, produces benefit in mouse models of systemic lupus erythematosus (SLE; Yin et al., 2015) and rheumatoid arthritis (RA; Abboud et al., 2018), with the addition of metformin augmenting its effect in SLE models. Combination therapy with 2‐DG, metformin, and 6‐diazo‐5‐oxo‐l‐norleucine (DON, an inhibitor of glutamine metabolism) prevents allograft rejection in transplantation models (Lee et al., 2015). 3‐bromopyruvate, an inhibitor of hexokinase and GAPDH, attenuates models of RA (Okano et al., 2017) and MS (Seki et al., 2017). Small molecule inhibitors of GLUT1 are effective in models of psoriasis (Zhang et al., 2018) and SLE (Li et al., 2019). Dichloroacetate (DCA), an inhibitor of PDHK1 that limits aerobic glycolysis and promotes OXPHOS by favoring pyruvate entry into the Krebs cycle rather than fermentation to lactate, has shown benefit in models of inflammatory bowel disease, RA, MS, and asthma (Bian et al., 2009; Gerriets et al., 2015; Ostroukhova et al., 2012). All the above models depend on aberrant activation of CD4 cells, and the benefit of the above pharmacologic agents was mediated at least in part by modulation of CD4 responses, including promotion of T_reg_ versus effector T cell differentiation and function. More recently, TEPP‐46, a small molecule targeting pyruvate kinase (PK) isoform PKM2, was shown to attenuate the experimental autoimmune encephalomyelitis (EAE) mouse model of MS by limiting the development of T_H_1 and T_H_17 cells (Angiari et al., 2019). PKM2 has moonlighting properties that include translocation to the nucleus after T cell receptor ligation and activation of pathways necessary for engagement of aerobic glycolysis, which were blocked by TEPP‐46.

Our own recent work identified inhibition of GAPDH and aerobic glycolysis as a key therapeutic mechanism of dimethyl fumarate (DMF), which is a clinically approved immunomodulatory drug used in the treatment of MS (Kornberg et al., 2018). Simply an electrophilic and cell‐permeable derivative of the Krebs cycle metabolite fumarate, the immunomodulatory properties of DMF were discovered serendipitously (Linker & Haghikia, 2016). Although it has been shown to alter the numbers and phenotypes of myeloid and lymphoid populations both in vitro and in vivo (Diebold et al., 2018; Ghadiri et al., 2017; Ghoreschi et al., 2011; Gross et al., 2016; Li et al., 2017; Longbrake et al., 2016; Michell‐Robinson et al., 2016; Smith, Calabresi, & Bhargava, 2018; Smith, Martin, Calabresi, & Bhargava, 2017; Spencer, Crabtree‐Hartman, Lehmann‐Horn, Cree, & Zamvil, 2015; Wu et al., 2017), its precise mechanism of action has remained uncertain. It has been well described to activate the NRF2 signaling pathway via electrophilic modification of KEAP1 (Linker et al., 2011), and its metabolite monomethyl fumarate produces immunologic effects via the hydroxycarboxylic acid receptor 2 (HCAR2; Chen et al., 2014)—important mechanisms that nonetheless fail to fully account for the in vitro and in vivo immunologic actions of the drug (Michell‐Robinson et al., 2016; Schulze‐Topphoff et al., 2016).

We found that DMF targets and inactivates GAPDH in both mice and humans through electrophilic attack on a critical active‐site cysteine, inhibiting glycolytic flux in activated macrophages and lymphocytes. The immunologic actions of DMF were mitigated by GAPDH overexpression or saturating concentrations of glucose, and replicated by the more specific GAPDH inhibitor heptelidic acid. Similar to glycolytic blockade with 2‐DG or HIF‐1α knockout, DMF reciprocally inhibited T_H_1 and T_H_17 survival, differentiation, and effector functions while promoting T_reg_ development, which is consistent with the clinical observation in patients that DMF induces lymphopenia selectively impacting effector cells while sparing T_reg_ cells and naïve T lymphocytes. By demonstrating that a clinically safe and effective immunomodulatory drug acts by inhibiting aerobic glycolysis, these findings support the clinical potential of targeting metabolism to regulate immune responses. Interestingly, Liao et al. (2019) recently showed that the endogenous metabolite itaconate and its derivative 4‐octyl itaconate, which are electrophilic compounds known to have anti‐inflammatory effects (Mills et al., 2018), also target GAPDH.

The kinetic properties of GAPDH suggest that it may be an ideal target for selective inhibition of Warburg glycolysis, in both cancer and autoimmunity. Several recent studies have shown that GAPDH, which is not a rate‐limiting enzyme under basal conditions, becomes rate‐limiting under Warburg conditions (Liberti et al., 2017; Shestov et al., 2014; Yun et al., 2015), which is consistent with our own finding that DMF inhibited glycolytic flux in activated, but not resting, macrophages. As such, GAPDH inhibition has the potential to selectively impact glycolysis in activated, inflammatory immune cells while sparing other tissues exhibiting normal energy metabolism. Consistent with this hypothesis, a therapeutic window for heptelidic acid was recently shown in a mouse model of breast cancer (Liberti et al., 2017). There are of course caveats to specifically targeting GAPDH. For instance, inhibition of GAPDH prevents ATP production from glycolysis and necessarily increases the concentration of upstream glycolytic metabolites, including G3P and DHAP. As described earlier, G3P and DHAP can be non‐enzymatically converted to the advanced glycation end‐product MGO, which in turn is metabolized to D‐lactate by glyoxalase enzymes. MGO, which increases with GAPDH inhibition, is a reactive intermediate that may serve dedicated signaling roles (Bollong et al., 2018; Gaffney et al., 2019; Galligan et al., 2018) but also is potentially toxic (Beisswenger, Howell, Smith, & Szwergold, 2003; Marín‐Hernández et al., 2016). d‐lactate derived from MGO can produce acidosis. The degree to which GAPDH inhibition predisposes to toxic effects from these byproducts remains uncertain. Interestingly, MGO‐derived modifications are enriched in glycolytic enzymes including GAPDH and appear to block glycolytic flux at the level of GAPDH (Gaffney et al., 2019), which may serve as a feedforward mechanism augmenting the effect of DMF on glycolysis.

Other clinically used drugs with immunologic actions may similarly target aerobic glycolysis. Teriflunomide, a dihydroorotate dehydrogenase inhibitor that limits T cell activation and is approved for treatment of MS, was recently shown to interfere with OXPHOS and aerobic glycolysis in activated T cells via functional inhibition of respiratory chain complex III (Klotz et al., 2019). Inhibitors of lactate dehydrogenase A (LDH‐A), which are under development for cancer, may find complementary roles as immunomodulators (Farabegoli et al., 2012; Rai et al., 2017).

Despite promising work in animal models and the discovery that some approved human therapies target glycolysis, it is important to note that the therapeutic window and long‐term safety of glycolysis inhibitors remain far from certain. Although many, if not most, differentiated tissues exhibit predominantly oxidative energy metabolism, critical exceptions exist. For instance, the brain is highly glycolytic and requires glucose in the absence of ketone bodies (Bélanger, Allaman, & Magistretti, 2011). As previously mentioned, many proliferative cell types depend on glycolysis, as well (De Bock et al., 2013; Folmes et al., 2011; Kondoh et al., 2007). Furthermore, under pathologic conditions such as ischemia, normally oxidative tissues may be forced to rely exclusively on glycolysis. Interestingly, DMF‐induced ketonuria (peaking 2 hr after each dose) has been reported in a patient with Type 1 diabetes mellitus (Krzystanek & Jarosz‐Chobot, 2018), suggesting that individuals with coexisting metabolic conditions might be particularly susceptible to adverse effects of glycolysis inhibition. The long‐term feasibility of pharmacologically targeting glycolysis in humans thus remains unknown and represents a major caveat to this approach as a treatment for autoimmune disease.

### 4.2 Dietary approaches

Metabolic pathways can be manipulated not only with pharmacologic agents, but also through dietary interventions (Table 1). The importance of glycolytic reprogramming in inflammatory responses suggests that glucose‐restricted diets, such as the ketogenic diet and modified Atkins diet, have potential benefit in inflammatory and autoimmune diseases. Ketogenic diet does, in fact, attenuate the course of EAE, with a concomitant decrease in markers of inflammation (Choi et al., 2016; Kim et al., 2012). In a small, randomized pilot trial in MS patients, 6 months of ketogenic diet led to improvements in patient‐reported physical and mental health outcomes compared to a control diet (Choi et al., 2016). Interestingly, glucose restriction with a ketogenic diet produced a decrease in patient lymphocyte counts relative to control diet, consistent with effects observed in patients taking DMF. Similar improvements in patient‐reported outcomes, namely depression and fatigue, were observed in an open‐label trial of the modified Atkins diet in MS patients (Brenton et al., 2019). It should be noted, however, that a ketogenic diet may produce immunologic actions through multiple mechanisms in addition to impaired glycolysis, such as via direct anti‐inflammatory signaling mediated by ketone bodies themselves. The ketone body β‐hydroxybutyrate, for instance, blocks NLRP3 inflammasome activation and has additional anti‐inflammatory actions via HCAR2 (Graff, Fang, Wanders, & Judd, 2016; Youm et al., 2015).

A number of other dietary strategies that include glucose restriction have been studied in animal models of autoimmunity and human patients with autoimmune disease, although many of these involve broad dietary restriction that makes isolating the importance of glucose difficult. For instance, continuous calorie restriction (CR) alters the immune response and improves neurologic outcomes in EAE (Esquifino et al., 2004, 2007; Piccio et al., 2008). Various forms of intermittent fasting (IF) and fasting‐mimicking diet (FMD) similarly produce benefit in EAE (Choi et al., 2016; Cignarella et al., 2018; Kafami et al., 2010), with a contribution from the gut microbiome (Cignarella et al., 2018). Short (7–10 day) periods of fasting have been evaluated in small, randomized studies of RA (Kjeldsen‐Kragh et al., 1991; Sköldstam, 1986) and MS (Choi et al., 2016), with improvement in patient‐reported outcomes. A randomized pilot study of CR and IF versus control diet in MS patients demonstrated the safety and feasibility of these diets over 8 weeks, with some improvement in patient‐reported emotional health outcomes (Fitzgerald et al., 2018).

Clearly, much more work is needed to fully understand the immunologic (and non‐immunologic) consequences of glucose restriction and other dietary interventions. Although based on a biological rationale with supportive evidence in animal models, much longer and larger clinical studies are required before any such dietary interventions should be recommended to patients, given the complexity of human physiology and the possibility of long‐term adverse effects.

## 5 CHALLENGES AND FUTURE DIRECTIONS

Despite the promise of targeting the unique glucose metabolism of activated immune cells to fine‐tune their behavior, many challenges and unanswered questions remain. One challenge particularly deserving of attention is the possibility that metabolic programs differ between cells studied in vitro versus in vivo. For instance, it has been reported that T_H_17 cells generated in vivo rely on OXPHOS rather than glycolysis (Franchi et al., 2017). Differences between cells generated in vitro versus in vivo have similarly been reported within other metabolic pathways and immune cell types, such as with serine metabolism in macrophages (Rodriguez, Ducker, Billingham, Weinberg, & Rabinowitz, 2019). Mechanistically, major questions remain as to why aerobic glycolysis is required for inflammatory immune responses, building on prior seminal studies. And finally, the gap between animal and human studies must be bridged to determine the true promise of immunometabolism in clinical disease—including the long‐term safety and feasibility of targeting essential pathways such as glycolysis. Pharmacologic and dietary studies in humans will ultimately determine whether metabolism can be targeted to produce selective modulation of the immune response without broadly toxic effects. The need for human data is of particular importance for dietary interventions, which have become popular among patients and the lay public despite a lack of evidence regarding safety and efficacy.

This review has focused on approaches for dampening immune responses in autoimmune disease. Attempts to exploit metabolic pathways to enhance tumor immunotherapy are much more complex, given the complicated interplay between the unique metabolic characteristics of tumors and infiltrating immune cells.

## 6 CONCLUSION

Initially thought to be unique to cancer cells, the Warburg effect, characterized by upregulated glycolysis with preferential fermentation of glucose to lactate even in the presence of oxygen, is observed in a wide range of inflammatory immune cells. Myeloid and lymphoid cells of both the innate and adaptive immune systems, ranging from lymphocytes and NK cells to DCs and macrophages, undergo Warburg‐like metabolic reprogramming in response to inflammatory stimuli. Blocking this upregulation of aerobic glycolysis dampens inflammatory responses, impairing the differentiation and function of pro‐inflammatory cell types while favoring anti‐inflammatory and regulatory immune phenotypes. The mechanisms underlying the requirement for glycolytic reprogramming remain incompletely understood. Common assumptions about the connection between aerobic glycolysis and anabolic biomass production or the kinetics of ATP production have not been definitively demonstrated. Several key mechanistic insights have been made, such as the role of the glycolytic enzyme GAPDH in regulating translation of cytokine mRNA and the importance of glycolytic enzymes and metabolites in regulating inflammatory transcriptional programs. Nonetheless, much work remains to fully understand why aerobic glycolysis is required for inflammatory immune functions.

There has been much interest in targeting aerobic glycolysis as a means of fine‐tuning the immune response to treat autoimmune diseases. Treatments targeting aerobic glycolysis take the form of both pharmacologic agents, such as inhibitors of glycolytic enzymes, and dietary interventions, including glucose‐restricted diets such as the ketogenic diet. A variety of pharmacologic inhibitors of glycolysis have shown promise in animal models of autoimmune disease, including 2‐deoxyglucose, inhibitors of the glucose transporter GLUT1, and dichloroacetate, which is an inhibitor of the enzyme PDHK1. DMF, an incompletely understood immunomodulatory drug used to treat psoriasis and MS, was recently shown to produce anti‐inflammatory effects through inhibition of GAPDH and aerobic glycolysis, providing proof of concept that targeting glycolysis is a viable strategy for treating human disease. GAPDH appears to play a unique regulatory role in Warburg glycolysis, making it an attractive target for selective modulation of glycolysis in activated immune cells without broad metabolic toxicity. Dietary strategies, including the ketogenic diet as well as restricted‐feeding diets such as CR and IF, have also shown benefit in animal models of autoimmunity and may have a role as adjunct therapy in humans. Although such diets have become highly popular among patients and the healthy lay public alike, evidence for the safety and efficacy of these diets in humans is lacking. Larger and longer studies are required before any of these diets should be recommended to patients.

## CONFLICT OF INTEREST

M.D.K. discloses that he has received consulting fees from Biogen, the company that manufactures dimethyl fumarate for use in multiple sclerosis.

## AUTHOR CONTRIBUTIONS

**Michael Kornberg:** Conceptualization; writing‐original draft; writing‐review and editing.

## RELATED WIREs ARTICLES

Computational approaches for understanding energy metabolism

Metabolic interactions in cancer: cellular metabolism at the interface between the microenvironment, the cancer cell phenotype and the epigenetic landscape

Metabolism in cancer metastasis: bioenergetics, biosynthesis, and beyond
